# Patterns of Limb and Epaxial Muscle Activity During Walking in the Fire Salamander, *Salamandra salamandra*

**DOI:** 10.1093/iob/obaa015

**Published:** 2020-05-27

**Authors:** S E Pierce, L P Lamas, L Pelligand, N Schilling, J R Hutchinson

**Affiliations:** 1 Museum of Comparative Zoology and Department of Organismic and Evolutionary Biology, Harvard University, Cambridge, MA 02139, USA; 2 Departamento de Clinica, Faculdade de Medicina Veterinária, Universidade de Lisboa, Av. da Universidade Técnica, 1300-345, Lisboa, Portugal; 3 Department of Comparative Biomedical Sciences, The Royal Veterinary College, Hawkshead Lane, North Mymms, Hatfield, AL9 7TA, UK; 4 Institute of Zoology and Evolutionary Research, Friedrich-Schiller-University Jena, Erbertstr. 1, Jena, 07743, Germany; 5 Structure and Motion Laboratory, Department of Comparative Biomedical Sciences, The Royal Veterinary College, Hawkshead Lane, Hatfield, AL9 7TA, UK

## Abstract

Salamanders and newts (urodeles) are often used as a model system to elucidate the evolution of tetrapod locomotion. Studies range from detailed descriptions of musculoskeletal anatomy and segment kinematics, to bone loading mechanics and inferring central pattern generators. A further area of interest has been *in vivo* muscle activity patterns, measured through electromyography (EMG). However, most prior EMG work has primarily focused on muscles of the forelimb or hindlimb in specific species or the axial system in others. Here we present data on forelimb, hindlimb, and epaxial muscle activity patterns in one species, *Salamandra salamandra*, during steady state walking. The data are calibrated to limb stride cycle events (stance phase, swing phase), allowing direct comparisons to homologous muscle activation patterns recorded for other walking tetrapods (e.g., lizards, alligators, turtles, mammals). Results demonstrate that *Salamandra* has similar walking kinematics and muscle activity patterns to other urodele species, but that interspecies variation does exist. In the forelimb, both the *m. dorsalis scapulae* and *m. latissimus dorsi* are active for 80% of the forelimb swing phase, while the *m. anconaeus humeralis lateralis* is active at the swing–stance phase transition and continues through 86% of the stance phase. In the hindlimb, both the *m. puboischiofemoralis internus* and *m. extensor iliotibialis anterior* are active for 30% of the hindlimb swing phase, while the *m. caudofemoralis* is active 65% through the swing phase and remains active for most of the stance phase. With respect to the axial system, both the anterior and posterior *m. dorsalis trunci* display two activation bursts, a pattern consistent with stabilization and rotation of the pectoral and pelvic girdles. In support of previous assertions, comparison of *Salamandra* muscle activity timings to other walking tetrapods revealed broad-scale similarities, potentially indicating conservation of some aspects of neuromuscular function across tetrapods. Our data provide the foundation for building and testing dynamic simulations of fire salamander locomotor biomechanics to better understand musculoskeletal function. They could also be applied to future musculoskeletal simulations of extinct species to explore the evolution of tetrapod locomotion across deep-time.

## Synopsis

**Padrones de actividad muscular epaxial y apendicular durante la cursorialidad de la salamandra-de-fuego, *Salamandra salamandra***Las salamandras y los tritones (urodelos) son utilizados con frecuencia como un sistema modelo para dilucidar la evolución de la locomoción en los tetrápodos. Los estudios previos varían de descripciones detalladas de la anatomía musculoesquelética y cinemática de los segmentos del cuerpo, a la mecánica de la capacidad de soporte de carga estructural ósea y la generación de padrones centrales. Otra área de interés ha sido los padrones de actividad muscular *in vivo*, medidos por electromiografía (EMG). Sin embargo, la mayoría de los trabajos anteriores con EMG se han centrado principalmente en los músculos de los miembros anteriores o posteriores en especies específicas o en el sistema axial de otras. En este trabajo, presentamos datos sobre los padrones de actividad muscular en los músculos de los miembros anteriores, posteriores y de la musculatura epaxial en una especie, *Salamandra salamandra*, durante la marcha continua. Los datos se calibran para los períodos del ciclo de caminar de los miembros (fase de soporte, fase de movimiento), lo que permite comparaciones directas con padrones de activación muscular homólogos registrados para otros tetrápodos cursoriales (por ejemplo, lagartos, caimanes, tortugas y mamíferos). Los resultados demuestran que *Salamandra* tiene padrones de cinemática cursorial y actividad muscular similares a otras especies de urodelos, pero con variación interespecífica. En los miembros anteriores, ambos los *m. dorsalis scapulae* y *m. latissimus dorsi* están activos en 80% de la fase de movimiento del miembro anterior, mientras que el *m. anconaeus humeralis lateralis* se activa en la transición de la fase de movimiento-soporte y permanece activo en 86% de la fase de soporte. En los miembros posteriores, ambos *m. puboischiofemoralis internus* y *m. extensor iliotibialis anterior* están activos en 30% de la fase de movimiento de los miembros posteriores, mientras que el *m. caudofemoralis* está activo durante el 65% de la fase de movimiento, permaneciendo activo durante la mayor parte de la fase de soporte. Con respecto al sistema axial, las porciones anterior y posterior del *m. dorsalis trunci* exhibe dos períodos de activación, un padrón consistente con la estabilización y rotación de la cintura pélvica y pectoral. Como sugirido anteriormente, la comparación de los tiempos de actividad muscular de *Salamandra* con otros tetrápodos cursoriales reveló similitudes en gran escala, lo que podría indicar la conservación de algunos aspectos de la función neuromuscular entre los tetrápodos. Nuestros datos proporcionan una base para la construcción y prueba de simulaciones dinámicas de la biomecánica locomotora de salamandras-de-fuego para comprender mejor las funciones musculoesqueléticas. Nuestros resultados también se pueden aplicar a futuras simulaciones musculoesqueléticas de especies extintas para explorar la evolución de la locomoción de tetrápodos en el tiempo profundo.

**Padrões de atividade muscular epaxial e apendicular durante a cursorialidade da salamandra-de-fogo, *Salamandra salamandra***Salamandras e tritões (urodelos) são freqüentemente utilizados como um sistema modelo para elucidar a evolução da locomoção em tetrápodes. Estudos anteriores variam de descrições detalhadas da anatomia musculoesquelética e cinemática dos segmentos corporais, a mecânica da capacidade de carga estrutural óssea e geradora de padrões centrais. Uma outra área de interesse tem sido os padrões de atividade muscular *in vivo*, medidos por eletromiografia (EMG). No entanto, a maioria dos trabalhos anteriores de EMG concentrou-se principalmente nos músculos dos membros anteriores ou posteriores em espécies específicas ou no sistema axial de outras. Nesse trabalho, apresentamos dados sobre os padrões de atividade muscular nos membros anteriores, posteriores e musculatura epaxial em uma espécie, *Salamandra salamandra*, durante caminhada em modo contínuo. Os dados são calibrados para os períodos do ciclo de caminhada dos membros (fase de apoio, fase de movimento), permitindo comparações diretas com padrões de ativação muscular homólogos registrados para outros tetrápodes cursoriais (por exemplo, lagartos, jacarés, tartarugas e mamíferos). Os resultados demonstram que *Salamandra* possui padrões de cinemática cursorial e atividade muscular semelhantes à outras espécies de urodelos, mas com variação interespecífica. Nos membros anteriores, ambos os *m. dorsalis scapulae* e *m. latissimus dorsi* estão ativos em 80% da fase de movimento do membro anterior, enquanto o *m. anconaeus humeralis lateralis* é ativado na transição da fase de movimento-apoio e continua ativo em 86% da fase de apoio. Nos membros posteriores, ambos *m. puboischiofemoralis internus* e *m. extensor iliotibialis anterior* estão ativos em 30% da fase de movimento dos membros posteriores, enquanto o *m. caudofemoralis* está ativo por 65% da fase de movimento, permanecendo ativo na maior parte da fase de apoio. No que diz respeito ao sistema axial, as porções anterior e posterior do *m. dorsalis trunci* exibe dois períodos de ativação, um padrão consistente com a estabilização e rotação da cintura peitoral e pélvica. Como préviamente sugerido, a comparação dos tempos de atividade muscular de *Salamandra* com outros tetrápodes cursoriais revelou similaridades em larga escala, potencialmente indicando a conservação de alguns aspectos da função neuromuscular entre tetrápodes. Os nossos dados fornecem uma base para a construção e testagem de simulações dinâmicas da biomecânica locomotora de salamandras-de-fogo para se entender melhor as funções músculo-esqueléticas. Nossos resultados também podem ser aplicados a futuras simulações músculo-esqueléticas de espécies extintas para explorar a evolução da locomoção de tetrápodes no tempo profundo.

## Introduction

Urodeles (salamanders and newts) are often considered to have retained the plesiomorphic tetrapod stance and gait and have long been used as a model system to elucidate the evolution of terrestrial locomotion (for a review, see Pierce et al. 2013). Their typically biphasic lifestyle (aquatic larva and terrestrial adult) has also been a source of inference in understanding the tetrapod water-to-land transition and the impact of environmental or ontogenetic shifts on locomotor performance (Ashley-Ross 1994a; Azizi and Landberg 2002; Wilson 2005; Landberg and Azizi 2010; Schoch 2014). Studies have detailed musculoskeletal anatomy (Francis 1934; Ashley-Ross 1992; Simons and Brainerd 1999; Walthall and Ashley-Ross 2006; Omura et al. 2015); limb and axial movements in both terrestrial and aquatic environments (Frolich and Biewener 1992; Ashley-Ross 1994a, 1994b; Gillis 1997; Ashley-Ross et al. 2009; Deban and Schilling 2009); limb and bone loading mechanics during terrestrial locomotion (Sheffield and Blob 2011; Kawano and Blob 2013; Kawano et al. 2016; Nyakatura et al. 2019); as well as the importance of the axial skeleton for coordinating activity patterns across disparate behaviors, including central pattern generators (e.g., Ijspeert et al. 2005; Ijspeert et al. 2007; Karakasiliotis and Ijspeert 2009; Cabelguen et al. 2010).

Another area of exploration has been *in vivo* muscle activity patterns during swimming and walking, as measured by electromyography (EMG). Such data on neuromuscular control of locomotion are valuable for helping to ascertain the functions of muscles, as anatomy alone may mislead about when muscles are used in certain behaviors. Coupling these data with other recordings such as limb/bone forces (Sheffield and Blob 2011; Kawano and Blob 2013; Kawano et al. 2016), muscle physiology (Ashley-Ross and Barker 2002) or musculotendinous length changes (Bennett et al. 1989) gives a more complete picture of how motion is achieved. Previous locomotor EMG studies on urodeles have given crucial insight into the diversity (or conservation) of activity patterns across the clade, among certain behaviors within species, and across muscles within a behavior (e.g., Frolich and Biewener 1992; Carrier 1993; Ashley-Ross 1995; Delvolvé et al. 1997; Deban and Schilling 2009; Ryczko et al. 2015). However, to date, sampling of muscle activity, and other locomotor mechanisms, has broadly been focused on a few species within the clades Ambystomatidae (mole and Coastal giant salamanders) and the Pleurodelinae (newts). As there are 600–700 species of extant urodeles with considerable ecological, morphological, ontogenetic, and locomotor diversity (Duellman and Trueb 1994), there is scope (and need) for greater taxonomic investigation.

The goal of this study was to document muscle activity patterns of the forelimb, hindlimb, and epaxial muscles during terrestrial walking in one species—the fire salamander *Salamandra salamandra* (Linnaeus 1758). Fire salamanders belong to the Salamandridae (Frost et al. 2006) and are remarkable among Urodela for their numerous terrestrial specializations, including variation of reproductive and developmental patterns from ovoviviparous to viviparous that provide them with increased independence from aquatic environments, including long dispersal distances (García-París et al. 2003; Bar-David et al. 2007; Buckley et al. 2007; Velo-Antón et al. 2015). These specializations contrast fire salamanders with other popular subjects of locomotion research such as highly aquatic newts (*Pleurodeles*, *Taricha*) or semi-terrestrial salamanders (*Ambystoma*, *Dicamptodon*). Further, most prior works examining muscle activity patterns in urodeles have focused on one specific part of the locomotor system—that is, mostly forelimb (e.g., *Triturus cristatus*, Szèkely et al. 1969) or hindlimb in specific species (e.g., *Ambystoma tigrinum*, Peters and Goslow 1983; *Dicamptodon tenebrosus*, Ashley-Ross 1995), and the axial system in others (e.g., *Ambystoma tigrinum*, Frolich and Biewener 1992; Bennett et al. 2001; *Ambystoma maculatum*, Deban and Schilling 2009; *Dicamptodon ensatus*, Carrier 1993)—meaning that our current understanding of urodele locomotor biomechanics is based on piecing together data across multiple species (Karakasiliotis et al. 2013).

To our knowledge, only one other study has examined forelimb, hindlimb, and epaxial muscle activity in a single taxon (Iberian ribbed newt, *Pleurodeles waltl*)—however, muscle activity data were all calibrated to the epaxial muscle; that is, “activity in the myomere located at 0.60 SVL level on the right side of the animal (‘0.60 SVL myomere’) was recorded as the reference point in the locomotor cycle” (Delvolvé et al. 1997, 639). Our study calibrates activity patterns for eight major appendicular and axial muscles with limb-specific stride cycle events to provide a dataset that is directly comparable with studies in the literature, including to other extant tetrapods. These data have at least three major benefits that we aimed to achieve here. First, a basic understanding of fire salamander terrestrial locomotion itself (and any hints of specialization relative to other species as noted above). Second, a comparative evolutionary analysis with similarly calibrated EMG patterns across Tetrapoda to test how conservative homologous muscle activity patterns are (see also Ashley-Ross 1995; Cuff et al. 2019). And third, to provide a basic dataset on activity patterns useful for testing the validity of future musculoskeletal simulations of locomotion in fire salamanders and urodeles more generally or even extinct tetrapods (e.g., Hicks et al. 2015; Rankin et al. 2016).

## Materials and methods

### Sample and housing

For this study, we sampled 10 adult fire salamanders, *S. salamandra*. Animals were partitioned into two experimental groups—five animals provided data for forelimb/anterior epaxial muscle EMG and five animals provided data for hindlimb/posterior epaxial muscle EMG. A summary of the body mass (g), total length (mm), and snout-vent length (mm) for each individual animal are given in Table 1. Salamanders were purchased from an animal supplier (Ameyzoo, Bovingdon, UK), housed in plastic containers laid with moist moss (using dechlorinated water), stored at 17°C daytime/13°C nighttime (12 h light/dark cycle), high humidity, and maintained on a diet of crickets. All procedures in the study were approved by the Royal Veterinary College’s Animal Welfare and Ethics Review Board (approval number AWERB-A-2013-5064) and conducted under a Home Office project license following the Animals (Scientific Procedures) Act of 1986 (UK).

**Table 1:** Summary of body dimensions and stride properties for each of the 10 *S. salamandra* individuals

Forelimb individuals	Sal_01	Sal_04	Sal_07	Sal_09	Sal_12	Mean
Body mass (g)	16	20	34	34	18	24.4 (4.0)
Total length (mm)	128	143	165	163	131	146.0 (7.8)
Snout-vent length (mm)	85	67	106	106	74	87.6 (8.0)
Total strides sampled	529	768	471	477	566	562 (54.34)
Stride duration (s)	0.78 (0.01)	0.69 (0.00)	1.06 (0.00)	0.76 (0.00)	0.91 (0.01)	0.84 (0.06)
Stance duration (%)	74.77 (0.20)	71.35 (0.23)	76.03 (0.14)	77.19 (0.15)	73.14 (0.16)	74.50 (1.03)
**Hindlimb individuals**	**Sal_06**	**Sal_08**	**Sal_11**	**Sal_13**	**Sal_19**	**Mean**
Body mass (g)	21	21	29	21	19	22.2 (1.55)
Total length (mm)	158	155	174	155	140	156.4 (5.4)
Snout-vent length (mm)	97	96	108	100	82	96.6 (4.2)
Total strides sampled	277	107	37	57	32	102 (45.72)
Stride duration (s)	0.98 (0.01)	0.83 (0.01)	1.91 (0.06)	1.94 (0.04)	1.17 (0.06)	1.04 (0.30)
Stance duration (%)	72.14 (0.19)	72.98 (0.46)	78.69 (1.04)	70.82 (0.69)	74.47 (0.51)	73.82 (1.35)

The individuals studied included five for forelimb and anterior epaxial muscle activity, and five for hindlimb and posterior epaxial muscle activity. Stance duration is represented as a percentage of total stride duration (i.e., duty factor*100). Variables are given as mean and standard error of the mean (s.e.m.). Mean stride duration and stance duration are the mean of the five individuals, thus representing a species mean. Numbers beside ‘Sal’ indicate the animal that participated in the study, not the total number of animals.

### Target muscles

**Fig. 1:**
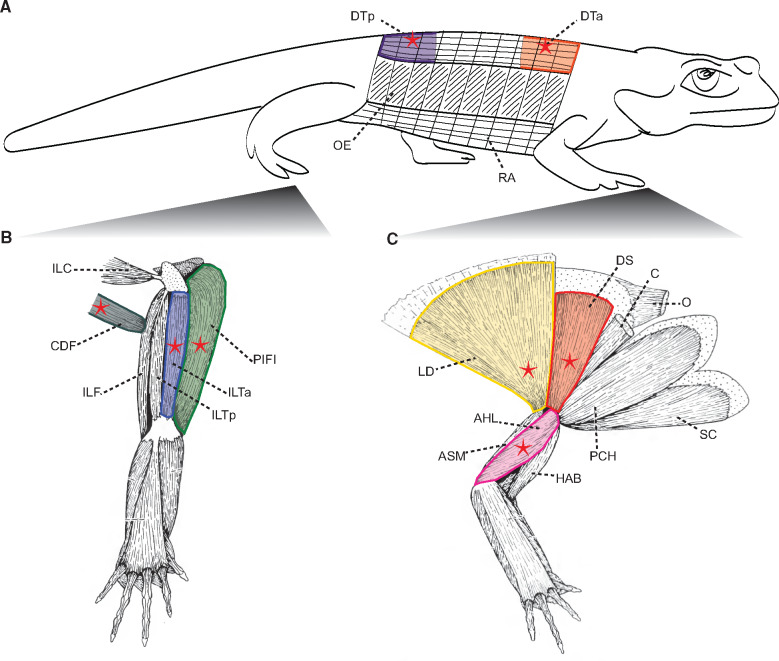
Postcranial muscular anatomy of *S. salamandra* showing placement of electrodes for recording muscle activity using electromyography. **(A)** Axial muscles showing placement of electrodes (red stars) for recording activity of the *m. dorsalis trunci anterior* (DTa) and *m. dorsalis trunci posterior* (DTp). **(B)** Hindlimb muscles showing placement of electrodes (red stars) for recording activity of *m. puboischiofemoralis internus* (PIFI), *m. extensor iliotibialis anterior* (ILTa), and *m. caudofemoralis* (CDF). **(C)** Forelimb muscles showing placement of electrodes (red stars) for recording activity of *m. anconaeus humeralis lateralis* (AHL), *m. dorsalis scapulae* (DS), and *m. latissimus dorsi* (LD). Abbreviations for remaining muscles depicted: ASM, *m. anconaeus scapularis medialis*; C, *m. cucullaris*; HAB, *m. humeroantebrachialis*; ILC, *m. iliocaudalis*; ILF, *m. iliofibularis*; ILTp, *m. extensor iliotibialis posterior*; O, *m. opercularis*; OE, *m. obliquus externus*; PCH, *m. procoracohumeralis*; RA, *m. rectus abdominus*; SC, *m. supracoracoideus*. Forelimb and hindlimb muscle illustrations are modified from Francis (1934).

We recorded the activity patterns of three forelimb and three hindlimb muscles, plus the anterior and posterior epaxial musculature (parts of *m. dorsalis trunci*) during steady state walking. Muscles were chosen based on presumed function, importance for controlling locomotion (e.g., size), as well as for ease of EMG implantation. Anatomical descriptions of muscles studied come from Francis (1934) and terminology from Walthall and Ashley-Ross (2006). Relative anatomical positions for each muscle are detailed in Fig. 1.

#### Forelimb muscles

*M. anconaeus humeralis lateralis* (AHL): The AHL is the most superficial upper forelimb muscle from a dorsal view. It originates from the anterolateral surface of the proximal portion of the humerus and inserts on the olecranon process of the ulna.

*M. dorsalis scapulae* (DS): The DS is a fan-shaped muscle that originates from the dorso-lateral surface of the cartilaginous suprascapula and inserts on the crista ventralis of the humerus via a strong tendon.

*M. latissimus dorsi* (LD): The LD is a fan-shaped, triangular muscle and is the largest of the dorsal shoulder muscles. It originates from dorsal fascia and extends from the DS posteriorly to cover three or four vertebrae. The LD crosses the shoulder joint and inserts on the posterior surface of the crista ventralis of the humerus via a strong tendon.

#### Hindlimb muscles

*M. puboischiofemoralis internus* (PIFI): The PIFI is the largest superficial dorsal muscle of the hindlimb. It originates from the internal (dorsal) face of the pubo-ischiadic plate and passes anterior to the ilium, crossing the hip joint. The PIFI inserts along the whole anterior face of the proximal femur.

*M. extensor iliotibialis anterior* (ILTa): The ILT originates from two separate heads (anterior and posterior) on the dorso-lateral surface of the ilium above the acetabulum. It crosses the hip and knee joint to insert via a long strong tendon on the tibial spine.

*M. caudofemoralis* (CDF): CDF is a deep, strap-like muscle with an oval cross-section. It originates on the transverse processes of the fourth and fifth caudal vertebrae and inserts via a strong tendon onto the crista ventralis of the femur.

#### Epaxial muscles

*M. dorsalis trunci anterior* (DTa) and *posterior* (DTp): The DT forms the bulk of the epaxial muscle mass and is segmented throughout the trunk with myosepta, which attach to neural spines and transverse processes of the vertebrae. Within and between successive myosepta, the DT muscle fibers run in a more or less sagittal (antero-posterior) direction.

### Surgery and electrode placement

We made hook electrodes from two strands of 0.025 mm diameter nichrome wire (A-M Systems #7615, Sequim, WA, USA). Insulation was removed from their tips; one end was bent into hooks and the other end soldered to a pin plug and sealed with epoxy which later was connected to the EMG recording equipment (see below). Before electrode implantation, we anesthetized the salamanders by partial immersion in an oxygenated 3 g/L aqueous solution of MS-222 (buffered to pH 7.0) for 20 min, beyond loss of righting reflex. During surgery, each salamander was gently wrapped in 2 g/L MS-222 soaked gauze and placed on its left side in a large petri dish, shallowly filled with 2 g/L MS-222 solution, connected to a constant supply of oxygen. The MS-222 solution was changed when pH drifted above 7.6. A heart rate monitor (Doppler with probe model 811-B, Parks Medical Electronics, Las Vegas, NE), was placed under the throat to monitor heart rate. As the surgery progressed, the concentration of MS-222 surrounding the animal was adjusted (concentration range 1–2 g/L), then gradually reduced such that the animal awoke from anesthesia soon after the surgery was completed (recovery time 31 ± 10 min). Buprenorphine 0.5 mg/kg (Vetergesic, Alstoe, York, UK) was administered intramuscularly for intraoperative analgesia. After surgery, salamanders were kept in damp plastic boxes (using dechlorinated water) and allowed to recuperate overnight. Postoperatively, all salamanders received a dose of 0.2 mg/kg of meloxicam orally (0.5 mg/mL oral suspension, Boehringer Ingelheim Animal Health UK Ltd, Bracknell, UK).

For the forelimb experimental group, we made an incision through the skin extending from the right anterior epaxial muscles anteroventrally, approximately between the DS and LD, and along the dorsal surface of the upper arm. For the hindlimb experimental group, we made an incision through the skin extending from the right posterior epaxial muscles posteroventrally, over the hip and along the thigh for approximately the length of the ILT. Following incision, target muscles were identified, and electrodes were inserted into the muscle belly using a 30-gauge hypodermic needle (see Fig. 1 for electrode positions). The skin incisions were sutured with 5-0 polyglatin 910 (Vycril, Ethicon, Johnson & Johnson, USA) such that the electrodes converged above the pectoral/pelvic girdle; electrode wires were glued together with cyanoacrylate to prevent snagging.

Following experimentation, salamanders were euthanized using a buffered 4–8 g/L aqueous solution of MS-222 (20 min) and pithed through the base of the skull. Electrode placement was confirmed via postmortem dissection under a binocular stereo microscope.

### Experimental setup

**Fig. 2:**
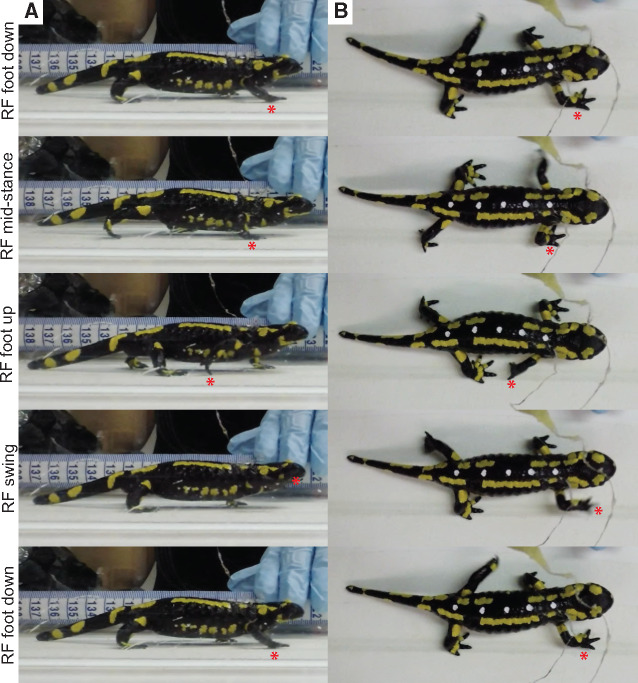
Right forelimb step cycle events during one stride in *S. salamandra*. **(A)** lateral view; and **(B)** dorsal view. Animal is walking on a treadmill at a constant speed. RF, right forelimb. Red star, tracks the right forefoot during the following step cycle events: foot down, mid-stance, foot up, swing, and foot down. For body dimensions and stride parameters of the animal depicted (Sal_01), see Table 1.

**Fig. 3:**
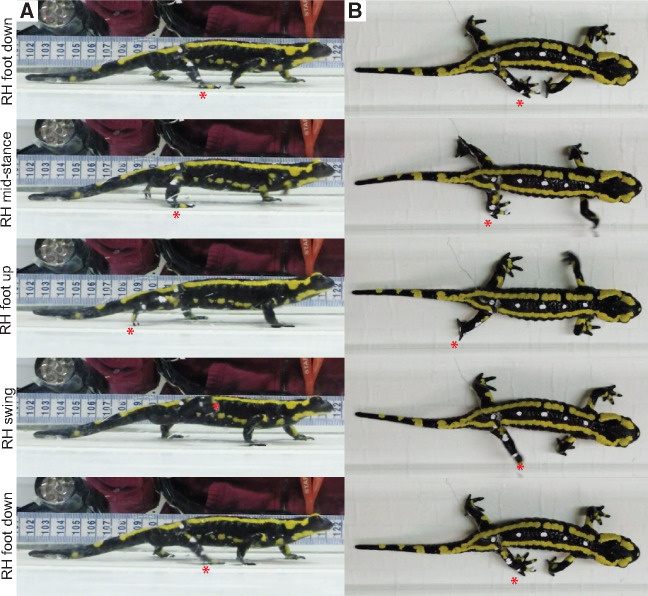
Right hindlimb step cycle events during one stride in *S. salamandra*. **(A)** lateral view; and **(B)** dorsal view. Animal is walking on a treadmill at a constant speed. RH, right hindfoot. Red star, tracks the right hindfoot during the following step cycle events: foot down, mid-stance, foot up, swing, and foot down. For body dimensions and stride parameters of the animal depicted (Sal_06), see Table 1.

Salamanders walked on a variable-speed, motor-driven treadmill (Panlab LE8710R) covered in masking tape to prevent slippage (Figs. 2 and 3). To constrain the salamanders to walk in a straight line, we mounted transparent plexiglass “walls” to the sides of the treadmill. One wall was fixed and had a tape measure attached to track positioning, while the other was adjustable in order to accommodate animals of different sizes and step widths. Prior to data collection, we adjusted the speed of the treadmill belt until each animal was walking comfortably at its preferred speed. During data collection, salamanders were kept moist with deionized water and were given regular breaks to prevent fatigue.

We recorded animals using two Hero 3+ GoPro cameras (San Mateo, CA) at a sampling frequency of 120 Hz, with cameras mounted to capture walking from the right lateral and dorsal perspectives. Cameras were triggered simultaneously before the beginning of a trial and allowed to run for 30 s, continuously. Once an animal was walking with a steady stride cycle, the EMG recording system was triggered. Electrode cables were connected to an Astro-med GRASS Rps312 RM amplifier, which interacted with a custom-made data acquisition (DAQ) box. The DAQ was, in turn, connected to a trigger box that simultaneously triggered a 10 s EMG recording and two LED lights placed within the video recording’s field of view for synchronization. The DAQ signal was passed through a custom LabView (National Instruments) script, which included a 10–400 Hz bandpass filter and a 50 Hz notch filter to reduce mains electrical noise. EMG signals were recorded at 5000 Hz, typically amplified 1000–10,000 times depending on signal quality.

### Data analysis

#### Kinematics

Before kinematic analysis, we removed “fisheye” artifacts from all GoPro video recordings using GoPro Studio “remove fisheye” function. Each video (which represents one trial) was also cropped to remove excess frames that fell outside the 10 s EMG recording period. After these adjustments, videos were manually digitized into strides using the DLTdv6() function (Hedrick 2008) in Matlab software (MathWorks, Natick, MA). As examining the relationship between stride cycle events and muscle activity was the primary purpose of this study, we digitized in right lateral view to determine stance and swing phases. Three events were digitized for each stride: foot contact, toe-off, and one frame before the following foot contact (Figs. 2 and 3). Only strides that represented steady walking at a constant speed were digitized (Table 2); these were determined qualitatively by a steady walking stride cycle prior to and after the digitized stride, as well as the animal remaining in the same relative position within the video frame. The number of digitized trials/strides per salamander is documented in Table 2. We also digitized the first frame when the LED lights switched on, allowing the video and EMG recordings to be synchronized.

**Table 2:** Summary of locomotion and EMG data collected and analyzed on *S. salamandra* forelimb and anterior epaxial muscles, and hindlimb and posterior epaxial muscles (subject info in Table 1)

Forelimb muscles			DTa	AHL	DS	LD
	#digitized trials	#digitized strides	#good signals	#good signals	#good signals	#good signals
Salamander_01	89	529	0	170	454	0
Salamander_04	103	768	459	672	711	659
Salamander_07	105	471	241	0	426	451
Salamander_09	91	477	0	0	320	137
Salamander_12	109	566	0	366	285	52
Total # trials/strides	497	2811	700	1208	2196	1299
Total # salamanders	5	5	2	3	5	4
Example EMG trace	—	—	Sal04_339	Sal12_347	Sal07_006	Sal07_191
**Hindlimb muscles**			**DTp**	**PIFI**	**ILTa**	**CDF**
	**#digitized trials**	**#digitized strides**	**#good signals**	**#good signals**	**#good signals**	**#good signals**
Salamander_06	88	277	102	100	150	178
Salamander_08	52	107	5	22	83	0
Salamander_11	29	37	13	31	35	10
Salamander_13	26	57	56	30	53	13
Salamander_19	13	32	32	32	31	8
Total # trials/strides	208	510	208	215	352	209
Total # salamanders	5	5	5	5	5	4
Example EMG trace	—	—	Sal19_006	Sal06_049	Sal06_003	Sal13_004

**Fig. 4:**
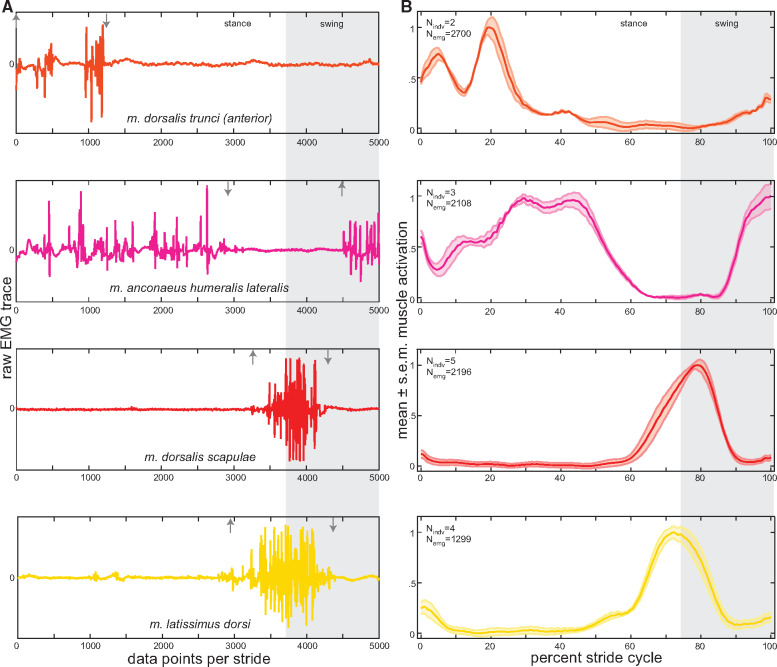
Forelimb and anterior epaxial muscle activity patterns during one stride. **(A)** representative raw EMG trace for each muscle (arrows indicate on/off); and **(B)** mean ± standard error of the mean (s.e.m.) muscle activity patterns for each muscle. Raw EMG traces were normalized to 5000 data points per stride and mean activity patterns were normalized to percent stride cycle. N_indv_, number of individuals; and N_emg_, number of EMG traces used to calculate the mean ± s.e.m. for each muscle. Further details of stride parameters and EMG data breakdown per animal can be found in Table 1 and Table 2, respectively.

“#good signals” refers to the number of EMG recordings that captured reliable muscle activity. “Example EMG trace” was randomly selected and is displayed in graphical form on Figs. 4 and 6. Abbreviations: DTa, *m. dorsalis trunci (anterior)*; AHL, *m. anconaeus humeralis lateralis*; DS, *m. dorsalis scapula*e; LD, *m. latissimus dorsi*; DTp, *m. dorsalis trunci (posterior)*; PIFI, *m. puboischiofemoralis internus*; ILTa, *m. extensor iliotibialis anterior*; CDF*, m. caudofemoralis*.

Qualitative inspection of the videos (Figs. 2 and 3) found that footfall and axial bending kinematics were as described for other terrestrial walking urodeles (see section “Results and discussion”). Considering our primary aim was to calibrate muscle activity with limb step cycle events, we do not provide a detailed quantitative analysis of joint kinematics here. However, we do provide a qualitative description of limb and body kinematics at major events in the stride cycle and augment this by approximating 2D limb segment angles from representative strides (measured using ImageJ software; https://imagej.nih.gov/ij/), including upper limb excursion, lower limb excursion, and lower limb inclination from the substrate (see Supplementary Fig. S1 for measurement protocol). Furthermore, we also compiled maximum limb segment excursion angles for other urodele species from the literature and broadly compared them to the approximated limb segment angles for *S. salamandra*. For a thorough review of urodele joint/segment kinematics, see Karakasiliotis et al. (2013).

#### Electromyography

Muscle activity patterns were analyzed in Matlab software (MathWorks, Natick, MA) using custom scripts. First, we plotted all raw EMG signals for each muscle and visually inspected them to determine quality; poor signals indicating electrode malfunction were removed from the data prior to further analysis. All remaining EMG signals were then rectified and filtered using a fourth order Butterworth 10–50 Hz bandstop filter, followed by a fourth order Butterworth 60 Hz lowpass filter to smooth the signal. This filtering process maximized signal to noise while achieving sharp time domain details. To compare between strides and between animals, stride duration was normalized to percent stride cycle and EMG signals resampled to 5000 data points. Next, each EMG signal was normalized to its maximum activation such that all signals ranged between 0–1. Each signal was then binned into 200 equal-sized bins with 25 data points per bin (or every 0.5% of the stride cycle) and then the average signal per bin determined. As each salamander had different numbers of “good” EMG signals per muscle, the mean muscle activity pattern for each individual was calculated before merging the data and determining the overall species mean and standard error of the mean (s.e.m.) muscle activity. Similarly, the mean percent stance phase (i.e., duty factor; stance time/stride time) for each individual was calculated and then merged to determine the overall species mean (s.e.m.) percent stance phase for the forelimb and hindlimb, separately (Table 1).

Furthermore, we determined the onset and offset of muscle activity for a random selection of ∼50 EMG signals per muscle using a modified script from Hodges and Bui (1996). The script uses two standard deviations above the base level (“resting”) as a threshold. As the method is very sensitive to noise, rectified EMG signals were smoothed using a “SmoothingFactor” of 0.5 prior to analysis. In addition to onset and offset, the script calculates burst duration, the rectified integrated area (RIA) under the curve (“intensity of muscle activation,” Loeb and Gans 1986; Ashley-Ross 1995), and RIA divided by burst duration (“relative force,” Ashley-Ross 1995). The mean (s.e.m.) for each variable per muscle was determined and is presented in Table 3. Onset, offset, and burst duration are expressed as percent stride cycle. Burst duration and RIA were normalized to 5000 data points and a maximum activation of 1.0, respectively.

**Table 3:** EMG summary variables measured for forelimb, hindlimb, and epaxial muscles of *S. salamandra*

		Onset	Offset	Duration	RIA	RIA/Dur	N_sample_
**Forelimb**							
	DTa	94.5 (6.7)	35.2 (3.8)	40.5 (2.0)	25.1 (1.0)	0.64 (0.01)	35,2
	AHL	79.9 (2.4)	64.1 (3.1)	83.9 (0.9)	44.1 (1.3)	0.52 (0.01)	45,3
	DS	54.5 (3.7)	96.3 (2.3)	41.7 (0.8)	24.1 (0.4)	0.58 (0.01)	50,5
	LD	52.8 (3.6)	91.4 (3.4)	38.6 (1.2)	24.2 (0.3)	0.65 (0.01)	39,4
**Hindlimb**							
	DTp	38.6 (2.1)	97.9 (3.3)	59.3 (1.4)	36.4 (0.9)	0.62 (0.01)	36,4
	PIFI	67.7 (1.9)	2.9 (2.6)	35.1 (1.0)	21.8 (0.2)	0.64 (0.01)	49,5
	ILTa	67.7 (2.3)	96.7 (3.7)	29.0 (1.2)	17.0 (0.4)	0.61 (0.01)	56,5
	CDF	82.9 (1.8)	59.3 (5.2)	76.4 (1.9)	17.9 (0.8)	0.23 (0.01)	26,4

Onset, Offset, and Duration are expressed in percent of stride length (from foot down to one frame before the subsequent foot down). Duration and RIA are normalized to 5000 data points and a maximum activity of 1.0, respectively. Thus, RIA/Duration (RIA/Dur) is also normalized and gives an approximation of relative “force.” All values (except *N*_sample_) are in mean (s.e.m.). *N*_sample_ provides the sample size (Strides, Individuals) used to calculate the summary variables. Abbreviations: DTa, *m. dorsalis trunci (anterior)*; AHL, *m. anconaeus humeralis lateralis*; DS, *m. dorsalis scapulae*; LD, *m. latissimus dorsi*; DTp, *m. dorsalis trunci (posterior)*; PIFI, *m. puboischiofemoralis internus*; ILTa, *m. extensor iliotibialis anterior*; CDF, *m. caudofemoralis*.

### Comparison with other tetrapods

To compare muscle activity patterns with other urodele species and tetrapod groups, we compared the data collected here with EMG data from the literature—taking inspiration from the work of Ashley-Ross (1995). Data were gathered from homologous muscles in animals moving with a terrestrial, quadrupedal walking gait, including: salamanders/newts (*Triturus cristatus*, Szèkely et al. 1969; *Dicamptodon tenebrosus*, Ashley-Ross 1995; *Pleurodeles waltl*, Delvolvé et al. 1997; *Ambystoma maculatum*, Deban and Schilling 2009); lizards (*Varanus exanthematicus*, Jenkins and Goslow 1983; *Sceloporus clarki*, Reilly 1995; *Chamaeleo calyptratus*, Higham and Jayne 2004); turtles (*Trachemys scripta*, Rivera and Blob 2010); alligators (*Alligator mississippiensis*, Gatesy 1997; Reilly and Blob 2003; Reilly et al. 2005); and mammals (*Felis catus*, English 1978; *Didephis virginiana*, Jenkins and Weijs 1979; *Rattus norvegicus*, Nicolopoulos-Stournaras and Iles 1984; *Canis familiaris*, Goslow et al. 1981; Carrier et al. 2008; Schilling and Carrier 2010). No primates were included in the comparison, even though some species walk quadrupedally. Muscle homologies followed Tsuihiji (2007) and Diogo et al. (2018). In order to compare across animals with different relative proportions of stance and swing phase, we normalized EMG onset and offset times using the conversion provided by Ashley-Ross (1995). If the muscle onset/offset occurred during the stance phase of the stride cycle, then:

$$X'=\frac{X}{\mathrm{stp}\%}*\mathrm{Sstp}\%$$

If, however, the onset/offset occurred during the swing phase of the stride cycle, then:

$$X^{'}=\frac{X-\mathrm{stp}\%}{\mathrm{swp}\%}*\mathrm{Sswp}\%+\mathrm{Sstp}\%$$

where, *X’* is the adjusted onset/offset time, *X* is the original onset/offset time, *stp%* is the percent stance phase of the animal (and limb) being adjusted, *swp%* is the percent swing phase of the animal (and limb) being adjusted, *Sstp%* is the mean percent stance phase for *S. salamandra* (forelimb or hindlimb), and *Sswp%* is the mean percent swing phase for *S. salamandra* (forelimb or hindlimb).

Step cycle events were not available or accurately reproducible for *Triturus* and *Pleurodeles*, so onset/offset was based on in-text qualitative description as they represent the only other studies that present forelimb muscle EMG data for urodeles.

## Results and discussion

### Stride cycle properties

A representative walking stride highlighting forelimb and hindlimb movements and axial bending in *S. salamandra* is shown in Figs. 2 and 3, and approximated maximum limb segment excursion angles can be found in Table 4. During steady state walking, the body is held clear of the substrate with only the tip of the tail in contact with the substrate. The footfall pattern is consistent with a lateral sequence gait and the body bends using a standing wave, as is typical for terrestrial walking salamanders (e.g., Hildebrand 1976; Edwards 1977; Karakasiliotis et al. 2013). In *Salamandra*, hindlimb stride duration (1.04 s) is slightly longer than forelimb stride duration (0.84 s); however, the percent stance phase (duty factor) is very similar between limbs (forelimb = 74.50% vs. hindlimb = 73.82%) (Table 1). These values are comparable to stride characteristics recorded for other salamander species using a walking gait: *Triturus cristatus* (Northern crested newt) has a forelimb duty factor of ∼75% (Szekley et al. 1969); *Dicamptodon tenebrosus* (coastal giant salamander) has a hindlimb stride duration between 0.97–1.14 s and duty factor between 68–72% (Ashley-Ross 2004a, 2004b; 2005); *Taricha torosa* (California newt) has a hindlimb duty factor of 77% (Ashley-Ross et al. 2009); *Ambystoma tigrinum* (tiger salamander) has a fore/hindlimb stride duration of 0.71/0.76s and a duty factor of 74/80% (Kawano and Blob 2013); and *Pleurodeles waltl* (Iberian ribbed newt) has a hindlimb stride duration of 1.52 s and a duty factor of ∼77% (Karakasiliotis et al. 2013) [with an axial bending duration of 1.11 s (Delvolvé et al. 1997)]. A duty factor >70% for walking salamanders is generally greater than walking in other “sprawling” tetrapods and quadrupedal mammals (Jenkins and Weijs 1979; Goslow et al. 1981; Higham and Jayne 2004; Starke et al. 2009; Baier and Gatesy 2013; Nyakatura et al. 2019).

**Table 4:** Comparison of maximum limb segment angles across urodeles during forward terrestrial walking

	*Ambystoma tigrinum*	*Ambystoma tigrinum*	*Dicamptodon tenbrosus*	*Pleurodeles waltl*	*Salamandra salamandra*	*Taricha torosa*
	Sheffield and Blob (2011)	Kawano et al. (2016)	Ashley-Ross (1994b)	Karakasiliotis et al. (2013)	this study	Ashley-Ross et al. (2009)
**Forelimb**						
Max protraction of upper arm	—	80	—	90	60	84
Max retraction of upper arm	—	130	—	142	140	126
Total protraction–retraction excursion of upper arm	—	50	—	52	80	42
Max rotation of upper arm	—	—	—	41	—	—
Max adduction of upper arm	—	80	—	83	<90	78
Max abduction of upper arm	—	110	—	128	>90	111
Max extension of elbow	—	130	—	119	142	147
Max flexion of elbow	—	75	—	79	60	63
Total extension–flexion excursion of elbow	—	55	—	30	82	85
Max extension of wrist	—	150	—	160	—	—
Max flexion of wrist	—	110	—	134	—	—
Max forearm-substrate	—	—	—	—	140	—
Min forearm-substrate	—	—	—	—	35	—
**Hindlimb**						
Max protraction of thigh	60	50	51	40	40	55
Max retraction of thigh	135	130	145	128	140	114
Total protraction–retraction excursion of thigh	75	80	94	88	100	60
Max rotation of thigh	—	—	—	66	—	—
Max adduction of thigh	78	75	—	89	<90	79
Max abduction of thigh	88	82	—	116	>90	117
Max extension of knee	150	145	173	155	150	159
Max flexion of knee	95	90	108	100	90	96
Total extension–flexion excursion of knee	55	55	65	55	60	63
Max extension of ankle	155	150	—	159	—	—
Max flexion of ankle	75	70	—	106	—	—
Max crus-substrate	—	—	132	—	120	—
Min crus-substrate	—	—	−5	—	20	—

Data were compiled from the literature and are presented as angles in degrees. Values given for *S. salamandra* are approximate (see text); those for the other species were taken directly from published tables/figures. All variables were transformed to reflect the measurement protocol used for *Salamandra* (see Supplementary Fig. S1). Note, values for *Ambystoma tigrinum* are for stance-only and forearm/crus-substrate angles for *Tarchia torosa* are not included as they were measured with a different (3D) protocol.

During forelimb movements in *Salamandra* (Fig. 2; Table 4), the right forefoot (RF) contacts the substrate just prior to the contralateral hindfoot contact and maximal trunk bending (i.e., concave on the left side of the body and convex on the right side of the body). At RF contact, the distal end of the upper arm is pointed anterolaterally, ∼60° from the direction of travel (viewed dorsally); the elbow is extended ∼100° (viewed dorsally) such that the foot is placed slightly laterally; the wrist is extended; and the forearm is inclined posteriorly ∼140° from the substrate (viewed laterally and in an anticlockwise direction). As the RF reaches mid-stance, the trunk is straight; the distal end of the upper arm points posterolaterally ∼110°; the elbow reaches maximum flexion of ∼45° such that the foot is placed medially; the wrist is flexed; and the forearm is inclined posteriorly ∼115° from the substrate. As the stride cycle progresses and the RF prepares for toe-off, the trunk bends toward the limb (i.e., concave on the right side of the body and convex on the left side of the body); the distal end of the upper arm is pointed posterolaterally ∼140° and is internally rotated; the elbow and wrist are extended; the foot is laterally placed; and the forearm is inclined anteriorly ∼35° from the substrate. During RF swing, the whole limb is raised above the level of the shoulder joint; the trunk straightens and then bends toward the contralateral limb (i.e., concave on the left side of the body and convex on the right side of the body); the limb externally rotates; and the elbow flexes in preparation for the following stride cycle.

When the right hindfoot (RH) of *Salamandra* makes contact with the substrate (Fig. 3; Table 4), the trunk is maximally flexed (i.e., concave on the right side of the body and convex on the left side of the body). The RH foot touches the substrate just posterior to the ipsilateral forefoot, with the toes pointed anteriorly. At RH contact, the distal end of the thigh is pointed anterolaterally ∼40° from the body wall (viewed dorsally); the knee is extended ∼150° such that the foot is placed lateral to the knee (viewed dorsally); and the crus is inclined ∼120° posteriorly from the substrate (viewed laterally and measured counterclockwise). As the RH limb reaches mid-stance, the trunk is straight; the distal end of the thigh is ∼90° from the body wall (i.e., pointing laterally); the knee is maximally flexed ∼90°; and the crus is anteriorly inclined ∼75°. As the RH approaches toe off, the distal thigh is pointed posterolaterally ∼140° from the body wall and is internally rotated; the ankle is extended; the crus is inclined anteriorly ∼20° from the substrate (measured in an anticlockwise direction); and the trunk is maximally flexed toward the contralateral hindlimb (i.e., concave on the left side of the body and convex on the right side of the body). During RH limb swing, the knee and ankle are fully extended; the thigh is externally rotated; the whole limb is raised above the level of the hip joint; the trunk straightens and then bends away from the RH (concave on right side of body) in preparation for the following stride cycle.

The limb (and trunk) segment kinematics of *Salamandra* are generally similar to those described for other salamander/newt species at comparable duty factors during terrestrial walking (Table 4; *Dicamptodon tenebrosus*, Ashley-Ross 1994b; *Taricha torosa*, Ashley-Ross et al. 2009; *Ambystoma tigrinum*, Sheffield and Blob 2011; Kawano et al. 2016). Although variation exists across species (and even within the same species but across different studies), maximum limb segment angles of *Salamandra* are within the range reported from previous studies. We do note one potential interesting pattern that may relate to lifestyle: *Salamandra* appears to have the greatest total protraction–retraction angular excursion of the upper arm and thigh compared to other species, which is primarily the result of greater segment protraction. This greater total upper limb segment excursion hints that *Salamandra* may have a relatively longer stride than other salamanders/newts—a potential adaptation for terrestrial locomotion and long dispersal distances (Bar-David et al. 2007). However, prior work has shown that ontogenetic stage and speed can have significant effects on kinematics properties in salamanders (Ashley-Ross 1994a) and that joint angular excursions are highly variable across tetrapods generally (Granatosky et al. 2019). More detailed analysis of joint kinematics and stride characteristics in *Salamandra*, and across urodele species with different lifestyles and locomotor ecologies, will help to pinpoint the source of kinematics variability and its correlates.

### Muscle activity patterns

#### Forelimb muscle EMG

**Fig. 5:**
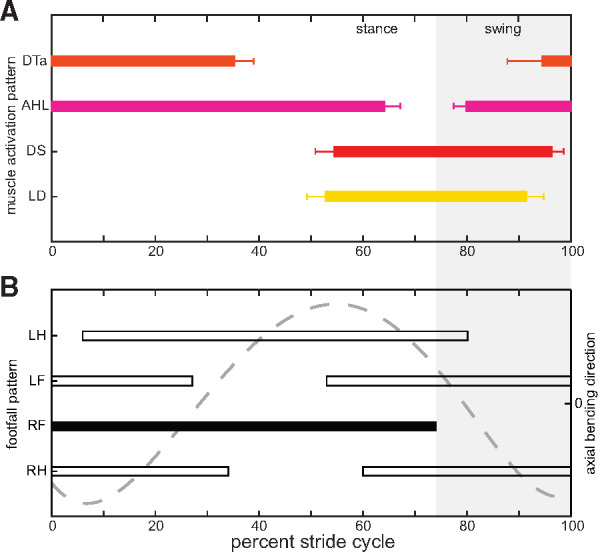
Forelimb and anterior epaxial muscle onset/offset in relation to limb and axial bending movements calibrated to the right forelimb step cycle. **(A)** Boxplots showing the mean ± standard error of the mean (s.e.m.) of onset and offset timings for each muscle; and **(B)** representative diagonal couplet lateral sequence footfall pattern (bars) and axial bending movements (dashed line) as seen in Figure 2. At right forelimb foot down (black bar), the body axis is convex toward the limb; during the latter part of stance, the body axis is concave toward the limb. A full summary of the EMG variables measured, including onset/offset timings can be found in Table 3. Abbreviations: AHL, *m. anconaeus humeralis lateralis*; DS, *m. dorsalis scapulae*; DTa, *m. dorsalis trunci (anterior)*; LD, *m. latissimus dorsi*; LF, left forelimb; LH, left hindlimb; RF, right forelimb; RH, right hindlimb.

During forelimb movements in *Salamandra*, the AHL is active for almost the entire stride cycle (Figs. 4 and 5). The onset of muscle activity occurs 80% into the forelimb stride cycle (∼21% into the swing phase) and continues until 64% of the next stride cycle (∼86% through the stance phase) (Table 3, Fig. 5). Therefore, the only time the AHL is not active is during the stance-swing phase transition; a period of time that is predicted to coincide with extension of the elbow joint (Fig. 2; Ashley-Ross et al. 2009; Karakasiliotis et al. 2013). The AHL has the longest duration (83.9%) and greatest RIA of all the muscles analyzed (44.1), but comparable RIA/Duration per stride cycle as the other muscles sampled (Table 3). Furthermore, the pattern of muscle activity is consistent with two bursts, one during the swing-stance phase transition and another encompassing the first two-thirds of the stance phase (Figure 4), a characteristic also noted in *Pleurodeles* (Delvolvé et al. 1997). Overall, this activity profile, in combination with qualitative observations of limb kinematics in *Salamandra* and quantitative joint kinematics described for other urodele species (e.g., Ashley-Ross et al. 2009; Karakasiliotis et al. 2013), indicates that the AHL functions primarily to counteract flexor moments at the elbow during stance, not to actively extend the elbow joint as has been previously proposed (Francis 1934; Walthall and Ashley‐Ross 2006). Active extension of the elbow joint during the stance-swing transition must be accomplished by alternative muscles, for example the *m. anconaeus scapularis medialis* or the *m. anconaeus humeralis medialis*; further EMG data are required.

Both shoulder muscles—DS and LD—have overlapping activity patterns (Figs. 4 and 5) and are active for similar durations (∼40% of the stride cycle; Table 3). Activity starts about halfway through the forelimb stride cycle (∼70% through the stance phase) and continues through ∼80% of the swing phase (Table 3, Fig. 5). Each muscle is characterized by one burst of activation, but they reach their peak activity at slightly different times: the DS peaks just after the onset of the swing phase, while the LD peaks just prior to the swing phase (Fig. 4). These are slightly different from data recorded for *Triturus* (Szèkely et al. 1969) and *Pleurodeles* (Delvolvé et al. 1997), in which the DS and LD are described as activating at toe-off and maintain activity during the entirety of swing phase. Delvolvé et al. (1997) also noted a transient secondary burst for each muscle during forelimb stance, which is not evident here. In *Salamandra*, both muscles appear to coordinate the transition from forelimb retraction to protraction; the LD activity peak is consistent with the final stages of forelimb retraction, while the DS activity peak coincides with limb elevation during the initial stages of forelimb protraction (Ashley-Ross et al. 2009).

#### Hindlimb muscle EMG

**Fig. 6:**
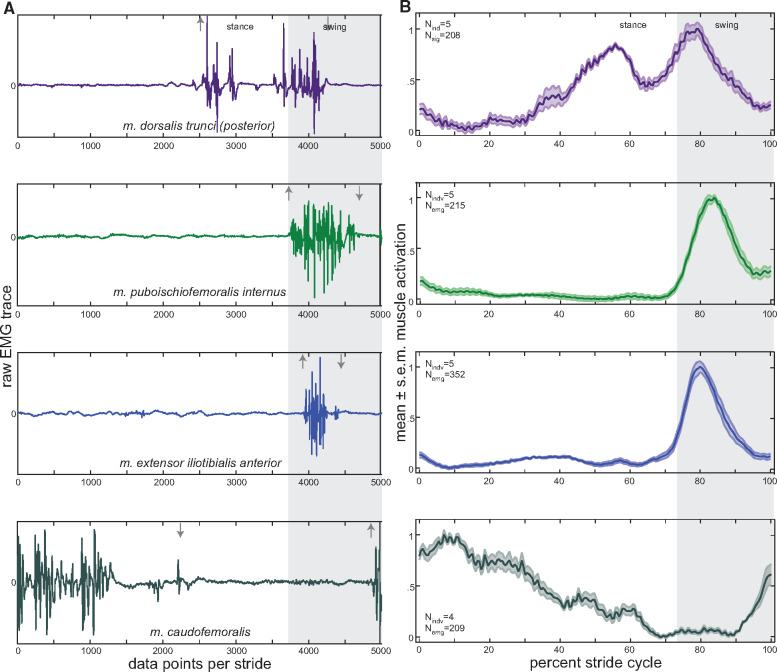
Hindlimb and posterior epaxial muscle activity patterns during one stride. **(A)** representative raw EMG trace for each muscle (arrows indicate on/off); and **(B)** mean ± standard error of the mean (s.e.m.) muscle activity patterns for each muscle. Raw EMG traces were normalized to 5000 data points per stride and mean activity patterns were normalized to percent stride cycle. N_indv_, number of individuals; and N_emg_, number of EMG traces used to calculate the mean ± s.e.m. for each muscle. Further details of stride parameters and EMG data breakdown per animal can be found in Table 1 and Table 2, respectively.

**Fig. 7:**
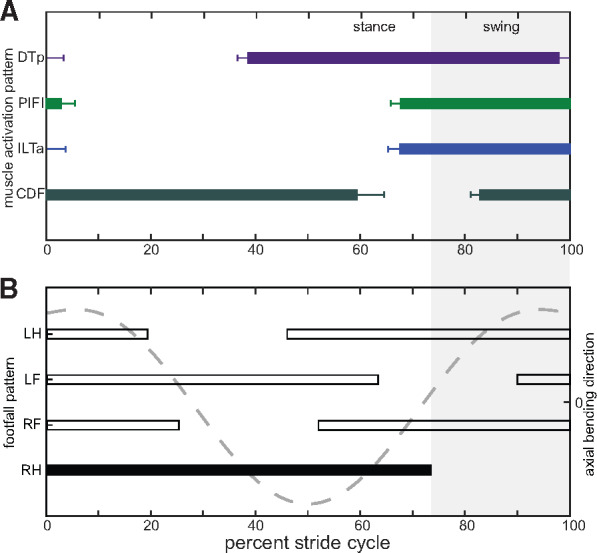
Hindlimb and posterior epaxial muscle onset/offset in relation to limb and axial bending movements calibrated to the right hindlimb step cycle. **(A)** Boxplots showing the mean ± standard error of the mean (s.e.m.) of onset and offset timings for each muscle; and **(B)** representative diagonal couplet lateral sequence footfall pattern (bars) and axial bending movements (dashed line) as seen in Fig. 3. At right hindlimb foot down (black bar), the body axis is concave toward the limb; during the latter part of stance, the body axis is convex toward the limb. A full summary of the EMG variables measured, including onset/offset timings can be found in Table 3. Abbreviations: CDF, *m. caudofemoralis*; DTp, *m. dorsalis trunci (posterior)*; LF, left forelimb; LH, left hindlimb; ILTa, *m. extensor iliotibialis anterior*; RF, right forelimb; RH, right hindlimb; PIFI, *m. puboischiofemoralis internus*.

During hindlimb movements in *Salamandra*, the PIFI and ILTa show overlapping bursts of activity during the swing phase of hindlimb movement (Figs. 6 and 7). Similar swing phase activity patterns for these muscles have also been noted in *Dicamptodon* (Ashley-Ross 1995) and *Pleurodeles* (Delvolvé et al. 1997). Both muscles in *Salamandra* activate 67.7% into the stride cycle (∼92% into stance phase), and peak just prior to mid-swing phase (Table 3, Fig. 6). Offset of muscle activity occurs toward the end of swing phase in the ILTa (96.7% of the stride cycle) and at the swing–stance phase transition in the PIFI (2.9% into the following stride cycle), resulting in the PIFI being active 6% longer than the ILTa (Table 3, Fig. 6). This pattern is the opposite of that described by Ashley-Ross (1995) for *Dicamptodon*. Additionally, Ashley-Ross (1995) found the PIFI to have the greatest RIA/Duration of all hindlimb muscles; while the PIFI of *Salamandra* displays similar RIA/Duration per stride cycle as the ILTa, forelimb muscles, and epaxials. Here a single burst of activation was recorded for both muscles (Fig. 6). Although a single burst was recorded in the ILTa of *Dicamptodon* (Ashley-Ross 1995), transient secondary bursts during stance characterized all other dorsal muscles of the hindlimb, including the PIFI. A secondary burst was also described for the PIFI of *Pleurodeles* (Delvolvé et al. 1997).

Although both the PIFI and ILTa are active during hindlimb protraction, they have slightly different presumed functions. The ILTa is often described as extending the knee joint (e.g., Francis 1934; Gatesy 1997; Reilly and Blob 2003; Higham and Jayne 2004; Reilly et al. 2005; Walthall and Ashley-Ross 2006; Cuff et al. 2019), but it is not active during knee extension in salamanders (Ashley-Ross 1995; Delvolvé et al. 1997; this study), a movement that occurs during the second half of stance. Instead, the ILTa appears to maintain the knee in a fully extended position during the stance–swing phase transition (as observed in our videos; see Fig. 3). Given its biarticular structure (crossing the hip and knee), the ILTa is also in an advantageous position to elevate (i.e., abduct) the leg during protraction, enabling the foot to clear the ground; and, in fact, this may be its primary function. The anatomical position of the PIFI, inserting on the anterior surface of the femur, makes it an effective protractor, with a secondary action in elevating the femur, as has been previously described (Ashley-Ross 1995; Delvolvé et al. 1997; Reilly and Blob 2003; Reilly et al. 2005).

In *Salamandra*, the CDF is active for just over three-quarters of the hindlimb stride cycle (76.4% duration; Table 3). The muscle is activated 82.9% into the stride cycle (∼65% into the swing phase) and deactivated 59.3% into the following stride cycle (∼80% into stance phase) (Table 3, Fig. 7). This activity profile agrees with *Pleurodeles* (Delvolvé et al. 1997) but differs from *Ambystoma tigrinum* and *Dicamptodon* in which the CDF becomes active ∼11–13% into stance (Peters and Goslow 1983; Ashley-Ross 1995). The CDF in *Salamandra* has one long burst of activation, peaking at the beginning of the stance phase and gradually decreasing toward the end of stance (Fig. 6). Irrespective of onset timing, a single CDF burst has been recorded in all other salamander species studied (Peters and Goslow 1983; Ashley-Ross 1995; Delvolvé et al. 1997). Although the CDF is active for a large portion of the stride cycle, second only to the AHL in the forelimb, its drawn-out profile results in reduced RIA/Duration (0.23) per stride cycle compared to the other muscles studied here (Table 3).

The CDF is considered a femoral retractor as it originates on the tail and attaches to the femur anteriorly via the crista ventralis (Francis 1934; Walthall and Ashley-Ross 2006). Retraction of the femur in salamanders is hypothesized to result (at least partially) from internal long-axis rotation of the femur as the CDF shortens, pulling the crista ventralis posteriorly and the animal forward over a flexed knee (“double crank”, Barclay 1946; Edwards 1977; Peters and Goslow 1983; Ashley-Ross 1994b). As femoral internal rotation has been shown to occur toward the second half of stance (Karakasiliotis et al. 2013), the CDF should activate after the stance phase has commenced and the knee has flexed. This predicted pattern of activity matches that found in *Ambystoma tigrinum* and *Dicamptodon* (Peters and Goslow 1983; Ashley-Ross 1995); however, it differs from the patterns evident in *Salamandra* and *Pleurodeles* (Delvolvé et al. 1997). In both of the latter taxa, the CDF activates prior to stance, indicating a secondary function. During stance, a flexor (dorsal/gravitational) moment is expected around the hip joint (Blob and Biewener 2001); as the CDF is oriented ventral to the femur during the beginning of stance in *Ambystoma* and *Pleurodeles* (femur is externally rotated, Karakasiliotis et al. 2013; Nyakatura et al. 2019), it may thus serve to counteract hip flexion (as predicted in *Alligator* by Gatesy 1997). We hypothesize that the CDF activates toward the end of swing in anticipation of resisting hip flexion and then continues to activate in order to retract and internally rotate the femur.

#### Epaxial muscle EMG

In *Salamandra*, the DTa becomes active at the end of the forelimb swing phase and continues to be active for ∼47% of the forelimb stance phase (or 35.2% of the stride cycle duration) (Table 3, Fig. 5). The DTp has a different activity pattern; starting roughly at hindlimb mid-stance (∼55% into hindlimb stance phase or 38.6% of the stride cycle duration) and continuing until the end of the swing phase (Table 3, Fig. 7). Thus, the DTa is primarily active during forelimb braking and limb loading and the DTp is primarily active during hindlimb propulsion and limb unloading. Although comparable data on terrestrial walking salamanders are not available (i.e., not calibrated to limb step cycle events), epaxial muscle activity during faster trotting (forelimb/hindlimb mean duty factor of 61%/64%) in *Ambystoma maculatum* has been quantified and shows a similar onset/offset pattern (Deban and Schilling 2009). The duration of muscle activity is ∼20% longer in the DTp and encompasses a greater integrated area; however, RIA/duration is comparable between the anterior and posterior parts of the muscle (Table 3), indicating they are capable of producing similar amounts of relative “force” for a given stride.

Both the anterior and posterior DT of *Salamandra* are also characterized by two activation bursts (Figs. 4 and 6). During DTa activity, the first burst reaches its peak ∼5% into the forelimb stride cycle (i.e., at maximum mid-trunk bending) and the second, larger burst reaches its peak ∼20% into the forelimb stride cycle (i.e., ∼28% of stance phase). Conversely, the first burst in the DTp is more drawn out and reaches its peak ∼57% in the hindlimb stride cycle (i.e., at maximum mid-trunk bending), with the second, larger (yet, more constrained) burst reaching its peak ∼78% into the hindlimb stride cycle (i.e., ∼15% into swing phase). If we assume that mid-trunk myomeres are active during maximum mid-trunk bending (as detailed by Delvolvé et al. 1997), the initial, smaller DT bursts recovered here would be in-phase with maximum mid-trunk bending and the larger, second burst would be out-of-phase (Figs. 4–7).

A similar biphasic activity pattern has been recorded previously in the anterior and posterior DT in some walking salamanders (Delvolvé et al. 1997) but not others (Deban and Schilling 2009). It has been suggested that the double burst may help to maintain the head/tail aligned to the direction of travel, to stabilize the trunk against extrinsic limb muscle action, and/or to stabilize and rotate the pelvis (Roos 1964; Ashley-Ross 1995; Delvolvé et al. 1997). By correlating DT muscle activity with limb positioning (this study), girdle kinematics (Ashley-Ross et al. 2009), and trunk twisting (Karakasiliotis et al. 2013), we provide a more nuanced interpretation for *Salamandra*. In the DTa, the first burst is hypothesized to stabilize the pectoral girdle during forelimb braking, while the second burst actively rotates the pectoral girdle toward the forelimb during limb loading and retraction and resists long-axis rotation of the trunk. Conversely, the first burst of the DTp stabilizes the pelvis during hindlimb propulsion, while the second burst of activity rotates the pelvis during hindlimb protraction and resists long-axis rotation of the trunk.

### Disparity across urodeles

At a broad comparative level, *Salamandra* does not seem to have especially different locomotor kinematics or neuromuscular specializations compared to other urodele species examined thus far. But, when viewed more precisely, our study does uncover some interspecies diversity across the clade. For instance, while limb and axial kinematics are wholly similar throughout the stride cycle, variation is present across species (Table 4). It is currently unclear what this variation is correlated with (e.g., phylogeny, ontogeny, environment, lifestyle, speed, measurement error), or if it reflects the less predictable nature of locomotion (Granatosky et al. 2019); more sophisticated kinematics techniques, such as X-ray Reconstruction of Moving Morphology (Brainerd et al. 2010), may help to more accurately investigate interspecies kinematics differences. In terms of EMG, in *Salamandra*, the LD and DS are active during the stance-to-swing phase transition, whereas they are described as swing phase-only muscles in newts (Szèkely et al, 1969; Delvolvé et al. 1997), and they lack a transient secondary burst as noted in *Pleurodeles* (Delvolvé et al. 1997). A single burst of PIFI activity was also noted here as well as divergent ILTa versus PIFI timings compared with *Dicamptodon* (Ashley-Ross 1995). Additionally, the CDF activates before stance in *Salamandra* and *Pleurodeles* (Delvolvé et al. 1997), unlike *Ambystoma tigrinum* or *Dicamptodon* (Peters and Goslow 1983; Ashley-Ross 1995). Thus, despite a similarly conservative sprawling quadrupedal gait and “plesiomorphic” morphology, there is evidence for kinematics and neuromotor variability within Urodela, which may relate to varying degrees of terrestrial specialization or other physiological/ecological factors. Future studies should examine whether morphological, biomechanical, behavioral, or other differences (such as measurement error) explain these patterns.

### Motor control evolution in tetrapods

In their comparison of shoulder muscle function between the Savannah monitor lizard (*Varanus exanthematicus*) and the Virginia opossum (*Didelphis virginiana*), Jenkins and Goslow (1983) hypothesized a set of “functional equivalences” between homologous muscles. Specifically, they noted that shoulder muscles had broadly similar activity timings with respect to step cycle and action irrespective of evolutionary anatomical transformation and interpreted this conserved motor pattern as being ancestral for tetrapods. Ashley-Ross (1994b, 1995) tested this idea by comparing hindlimb terrestrial walking kinematics and muscle activity timings in *Dicamptodon* to a broad range of amniotes with different limb postures (lizards, crocodiles, birds, mammals). She predicted homologous muscles would share similar activity periods during a common set of hindlimb step cycle events. Although most ventral muscles studied did share similar activity timings, supporting neuromuscular conservatism, some dorsal muscle motor outputs were more variable across tetrapod species.

**Fig. 8:**
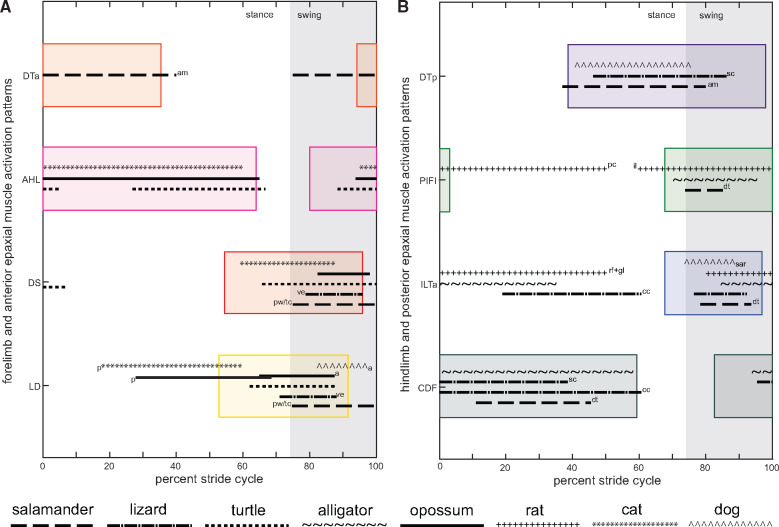
Comparison of muscle onset/offset timings across tetrapods. **(A)** Forelimb and anterior epaxial muscles; and **(B)** hindlimb and posterior epaxial muscles. Colored boxes represent the mean onset/offset timings for *Salamandra salamandra*. Muscle onset/offset timings for all animals were calibrated to the limb step cycle (duty factor) of *Salamandra salamandra.* Abbreviations: a, anterior part of LD; am, *Ambystoma maculatum*; dt, *Dicamptodon tenbrosus*; cc, *Chamaeleo calyptratus*; il, *m. iliacus*; p, posterior part of LD; pc, *m. pectineus*; pw, *Pleurodeles waltl*; rf+gl, *m. rectus femoris + gluteus maximus*; sar, *m. sartorius*; sc, *Sceloporus clarki*; tc, *Triturus cristatus*; ve, *Varanus exanthematicus*. See Material and methods for all species examined and for references. See Figs. 5 and 7 for muscle abbreviations on the Y-axis.

The results from our hindlimb muscle cross-clade comparison seem to concur with Ashley-Ross (1995): the CDF shows broadly similar activity timings across tetrapods, while the PIFI and particularly the ILTa (and their homologs) are more variable (Fig. 8B). While most species show ILTa/PIFI activity during the swing phase like *Salamandra*, some also show activity during stance. However, it is unclear whether this variation is due to species differences (i.e., mammal homologs are differentiated into multiple muscles, perhaps increasing functional diversification), speed variation, or potential confounding factors such as electrode placement or measurement error. Cuff et al. (2019) studied 13 different appendicular muscles in birds and crocodiles, of which only the *m. iliotibialis* has partial homology with any of those measured here (i.e., the ILTa) and found similar activity timings: late swing-early stance but timed to coincide with active knee extension in archosaurs; unlike in salamanders. Thus, conserved activity patterns may be used in different ways, depending on joint morphology and kinematics.

Tetrapod forelimb muscle activity timings do appear to share similarities as noted by Jenkins and Goslow (1983). Of the three forelimb muscles that we studied (Fig. 8A), the AHL and DS are directly comparable, with very little variation between species. The LD does show more variation, but this is probably due to electrodes being placed in the anterior versus posterior part of the muscle. Jenkins and Weijs (1979) noted that the anterior and posterior parts of the LD in *Didelphis* were active at different points in the stride cycle and classified them into different functional groups. The LD activity patterns that we recorded for *Salamandra* align with the anterior motor output of both the opossum and the dog—bridging the stance–swing phase transition. The conserved neuromuscular control of the forelimb is also reflected in the epaxial muscles, especially the DTp where multiple species comparisons could be made (Fig. 8A). In this case, both “sprawling” (salamanders, lizards) and “upright” (mammals) tetrapods have directly overlapping activity timings, even though the axial skeleton moves in dramatically contrasting ways.

Combined, these data reveal additional broad-scale neuromuscular conservatism across tetrapods that may indicate acquisition from a common ancestor. However, some smaller-scale differences exist highlighting the need for more comparative studies with greater taxonomic and muscular breadth, as well as deeper exploration of the underlying mechanisms that generate motion. Much as our study reveals instances where morphology-based predictions of muscle functions can be inaccurate (Lauder 1995), EMG patterns are only one aspect of locomotor behavior. Dynamic simulation of locomotor function in salamanders could predict muscle activity patterns from experimentally measured biomechanics, allowing quantitative tests of muscle and joint functions, as well as comparisons with the EMG patterns here for verification and validation purposes (Hicks et al. 2015; Rankin et al. 2016). Such data could be applied to musculoskeletal simulations of extinct animals to explore the evolution of locomotion performance across deep-time.

## Supplementary Material

obaa015_Supplementary_Data
