# Supplementary figures and images for: Patterns of Limb and Epaxial Muscle Activity During Walking in the Fire Salamander, Salamandra salamandra
Source: Integr Org Biol. 2020 May 27;2(1):obaa015. doi: 10.1093/iob/obaa015 (PMC7671131; doi:10.1093/iob/obaa015)

**
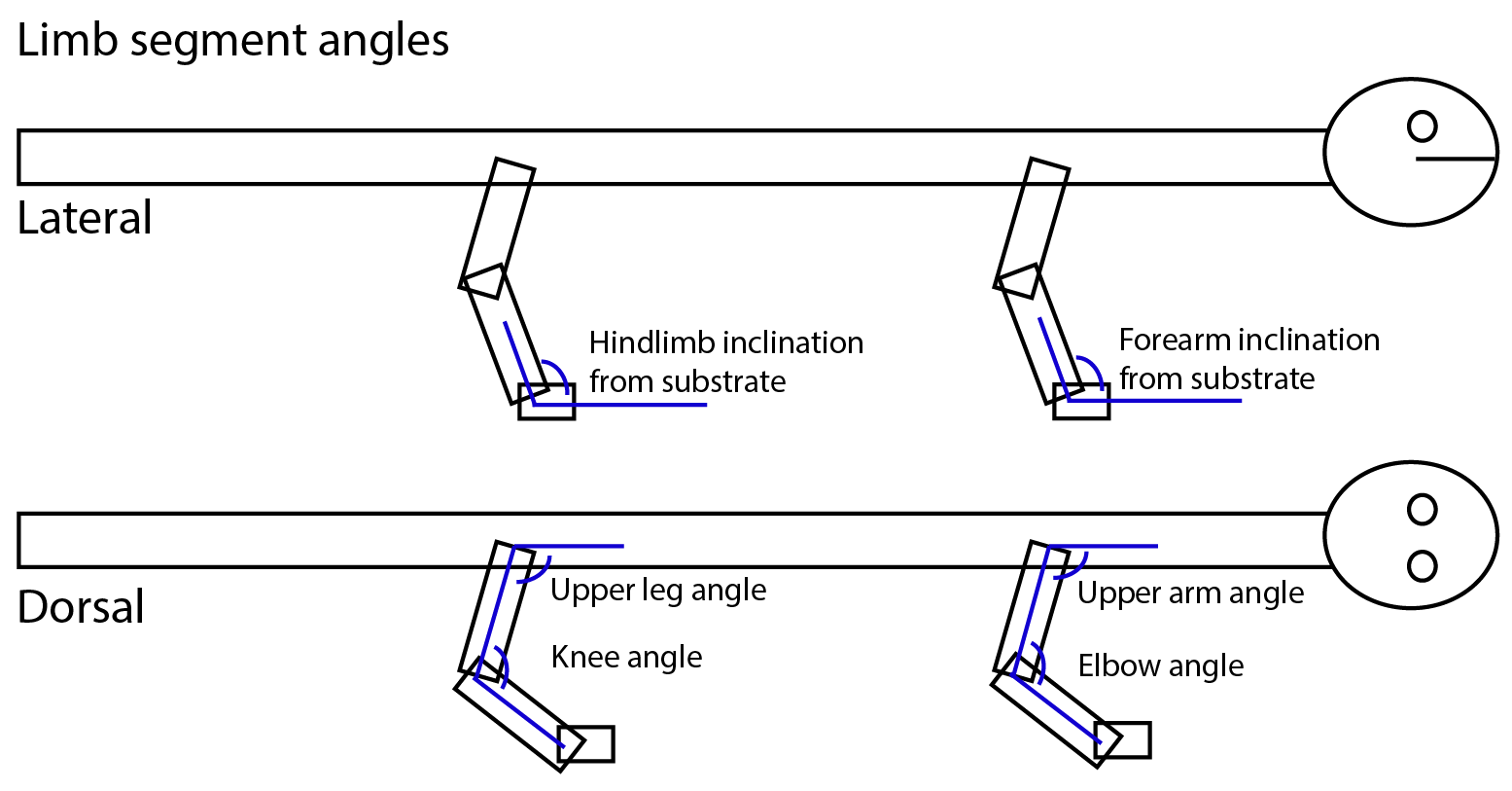
**

**Supplementary Figure S1.** Limb segment measurement protocol.

Supplement: obaa015_Supplementary_Data [file obaa015_supplementary_data.docx]
